# Effects of Heavy Metals on Stomata in Plants: A Review

**DOI:** 10.3390/ijms24119302

**Published:** 2023-05-26

**Authors:** Zhaolai Guo, Yuhan Gao, Xinqi Yuan, Mengxiang Yuan, Lv Huang, Sichen Wang, Chang’e Liu, Changqun Duan

**Affiliations:** 1 School of Ecology and Environmental Science, Yunnan University, Kunming 650091, China; 18314522882@163.com (Z.G.); gaoyuhan83@163.com (Y.G.); xqyuan2022@163.com (X.Y.); ymx2045024635@163.com (M.Y.); moanna@gmail.ynu.edu.cn (L.H.); wangsichen0101@163.com (S.W.); change@ynu.edu.cn (C.L.); 2 Yunnan Key Laboratory of Plateau Ecology and Degraded Environment Restoration, Kunming 650000, China

**Keywords:** soil pollution, air pollution, guard cells, structure of stomata, toxicity mechanisms

## Abstract

Stomata are one of the important structures for plants to alleviate metal stress and improve plant resistance. Therefore, a study on the effects and mechanisms of heavy metal toxicity to stomata is indispensable in clarifying the adaptation mechanism of plants to heavy metals. With the rapid pace of industrialization and urbanization, heavy metal pollution has been an environmental issue of global concern. Stomata, a special physiological structure of plants, play an important role in maintaining plant physiological and ecological functions. Recent studies have shown that heavy metals can affect the structure and function of stomata, leading to changes in plant physiology and ecology. However, although the scientific community has accumulated some data on the effects of heavy metals on plant stomata, the systematic understanding of the effects of heavy metals on plant stomata remains limited. Therefore, in this review, we present the sources and migration pathways of heavy metals in plant stomata, analyze systematically the physiological and ecological responses of stomata on heavy metal exposure, and summarize the current mechanisms of heavy metal toxicity on stomata. Finally, the future research perspectives of the effects of heavy metals on plant stomata are identified. This paper can serve as a reference for the ecological assessment of heavy metals and the protection of plant resources.

## 1. Introduction

“Heavy metals” is a general term for metals and metalloids with an atomic number above 20 and a relative density greater than 4 g/cm^3^. Common heavy metals include lead (Pb), cadmium (Cd), copper (Cu), zinc (Zn), chromium (Cr), arsenic (As), and mercury (Hg), etc. [1,2]. With the rapid development of industrialization and urbanization, human activities lead to the release of heavy metals into the environment through various pathways. Heavy metal pollution has become a global environmental issue because of its long residual time in the environment and its non-degradable physical and chemical properties. An increasing amount of studies have confirmed that heavy metals can cause a series of negative environmental and ecological impacts [3,4,5].

**Figure 1:**
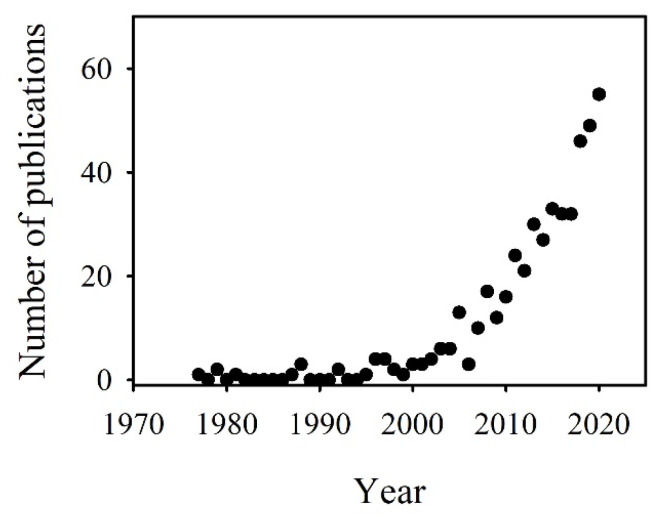
Number of articles published between 1970 and 2020 involving stomatal effects. The data are based on the use of “stomata” and “heavy metal” as keywords and were retrieved from the Web of Science.

Stomata are the specialized pores in the epidermis of plant cells, and are involved in the implementation of these functions (e.g., photosynthesis, respiration, and transpiration). Given their direct contact with the external environment, stomata are considered to be an important apparatus for plants to adapt to environmental stress [6,7,8,9]. An increasing number of studies have shown that heavy metal exposure can cause damages in the structure and function of plant stomata, and ultimately lead to changes in plant physiology and ecology [4,10,11,12]. Our analysis revealed an increasing interest in the study of stomata under heavy metal stress (Figure 1); the systematic understanding of the effects of heavy metals on plant stomata is still limited because research on the interactions between heavy metals and stomata is still in its infancy. Thus, the purpose of this work is to systematically understand the effects of heavy metals on plant stomata. We have reviewed the progress of the current research on the effects of heavy metals on plant stomata, including the effects and mechanisms of toxicity at the apparent, cellular, and molecular levels, and have outlined the areas of research that need to be expanded and deepened. It is anticipated that this paper will contribute to a better understanding of plant stomatal function and its interaction with heavy metals, and thus provide a reference for the subsequent evaluation of plant tolerance or adaptation to heavy metal stress.

## 2. Structure and Function of Stomata

**Figure 2:**
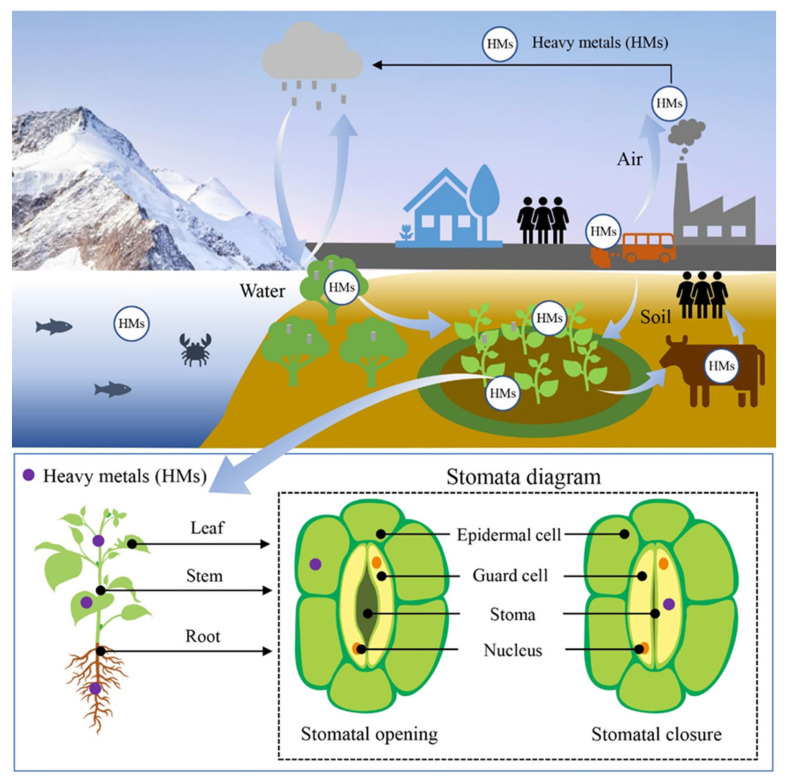
Sources of heavy metals in plant stomata. There are three main sources of heavy metals in plant stomata: the air, soil, and water cycles. Heavy metals can be enriched on the plant surface through atmospheric deposition, and thus enter the stomata. They can also enter the stomata through the uptake of surface water and soil nutrients by the plant roots.

**Figure 3:**
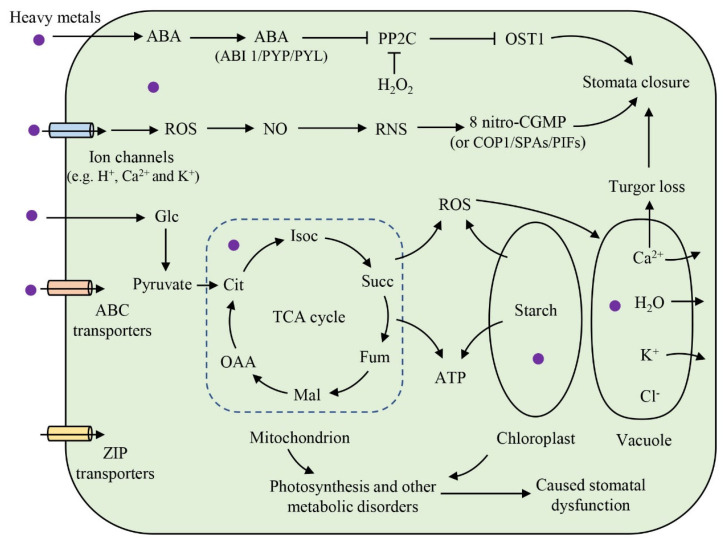
Schematic diagram of the toxic effects and mechanisms of heavy metals on stomata. Heavy metals can induce an increase in ABA (abscisic acid) and ROS (reactive oxygen species), which can lead to stomatal closure. Moreover, heavy metals can affect stomatal photosynthesis and energy metabolism disorders by damaging the structure and function of mitochondria and chloroplasts, ultimately causing stomatal dysfunction. Glc: glucose; TCA: tricarboxylic acid; Citric acid: citrate; IsoC: isocitrate; 2−OG, 2−ketoglutarate; Succ: succinate; Fum: fumarate; Mal: malic acid; OAA: o −aloacetate; Glutamate: glutamic acid; Glutamine: glutamine.

Stomata are specialized apparatus in plants, and play a vital role in maintaining plant growth and development [13]. To create stomata, epidermal cells first differentiate to form meristemoid mother cells (MMC), which are then divided asymmetrically to create meristemoid cells (MC), and finally differentiated once more to form guard mother cells (GMC). Two kidney-shaped guard cells are formed by the symmetrical division of GMCs, which eventually form a stomatal complex (Figure 2) [14,15,16]. Guard cells can regulate the behavior of stomata by ion-driven expansion, opening or closing, resulting in higher photosynthetic efficiency [17]. In addition, the transpiration of water is controlled by the regulation of the micro-pores of stomata [18]. Stomata differ markedly in structure and function in different plant species, possibly as a result of evolutionary adaptation to different environmental changes in different plants [19,20,21,22,23]. Stomata play a very important role in plant growth and development, and thus there are many studies on stomata under different conditions, such as those mediated by carbon dioxide, those under drought stress, those mediated by high and low temperatures, those mediated by care, and those mediated by phytohormones that regulate stomata (Figure 3) [23,24,25,26,27,28,29].

## 3. Role of Stomata in Heavy-Metal-Induced Plant Damage

Stomata are key tissue structures or organs in plants that respond to heavy metal stress. An increasing number of studies have shown that heavy metals can be enriched in plants through biogeochemical cycles, thereby causing damage to the stomatal morphology and structure, and ultimately interfering with several physiological metabolic processes in plants (Figure 2). Cd can inhibit photosynthesis in wolfsbane by reducing stomatal conductance and causing a reduction in carbon dioxide uptake [5]. Cd can increase the availability of intracellular CO_2_ by increasing stomatal conductance and pore size in mustard, leading to an increase in photosynthetic rate [30]. Stomatal resistance of silver maple seedlings is reduced when stimulated by Cd at low concentrations, thereby increasing transpiration; when Cd concentration exceeds a certain value, stomatal resistance increases or stomata close, thereby reducing transpiration intensity [31]. Pb affects plant transpiration by reducing stomatal conductance, and thus the transpiration rate in wheat [32]. Cd can improve the water use efficiency of soybean by increasing the stomatal area and decreasing the stomatal size [33]. These results suggest that changes in stomatal morphological and structural characteristics play an important role in heavy metal-induced changes in the plant physiological metabolism. Therefore, the elucidation of the stomatal damage effect and its mechanism is essential for understanding the stress mechanisms of plants in response to heavy metals.

## 4. Toxic Effects and Mechanisms of Heavy Metals on Plant Stomata

### 4.1. Effect of Heavy Metals on Stomatal Behavior

The effects of heavy metals on different plants are different, and many studies of heavy metal-induced plant stomatal closure have been conducted (Table 1). Lead can induce stomatal closure in tobacco, *Leucaena leucocephala*, black gram, and soybean plants [11,34,35,36]; Cd can induce the stomata closure of *Monochoria hastata*, rice, *Brassica juncea*, *Calophyllum brasiliense* Cambess, cowpea, *Hordeum Vulgare*, and *Pennisetum* sp. [5,37,38,39,40,41,42]; Hg can induce spruce stomata to close [43]; Zn can induce stomata closure in cowpea plants [12]; Cu and Ni can induce tomato stomata to close [44,45]; Ba can induce soybean stomata to close [17]; Sb can induce stomata closure in *Acorus calamus* [46]; and Al exposure can lead *Quercus glauca* Thumb plant stomata to close [47]. These results suggest that different heavy metal exposures can lead to stomatal closure in different plant species, which is likely to be one of the compensatory mechanisms by which plants respond to heavy metal stress.

Although an increasing number of studies have shown that exposure to heavy metals can lead to stomatal closure, the causes of stomatal closure are not fully understood. A previous study has suggested that heavy-metal-induced stomatal closure may be linked to changes in plant abscisic acid (ABA) levels, which is a plant hormone, also known as a “stress hormone”, that plays an important role in the regulation of stomata [48]. Heavy metal stress has been shown to increase ABA levels in plants, which causes water loss in guard cells and thus promotes stomatal closure [48]. Another study has shown that the stomatal closure caused by heavy metals was not related to the change of ABA content, and heavy metals can cause an ion imbalance by affecting the calcium channel of guard cells, thereby changing stomatal behavior [49]. A recent study showed that heavy metals may also be transported via plant root uptake to stomatal guard cells for direct interaction with stomata, thus causing stomatal closure. Clearly, the causes of heavy metal-induced stomatal closure in plants are extremely complex, which may be related to the concentration and duration of heavy metal exposure. When plants are exposed to low concentrations of metal stress for a short period of time, the increase in stomatal resistance leads to changes in stomatal behavior. When plants are exposed to low concentrations of metal stress for a long period of time, this leads to an increase in ABA, which in turn causes stomatal closure. However, when plants are subjected to high concentrations of metal stress, this can lead to wilting and water-passive stomatal closure.

**Table 1:** Effects of heavy metals on stomata.

Heavy Metal	Processing Time and Concentration	Plant Species	Plant Type	Toxic Effect	References
Cd	50, 100 mg/kg; 3 months	*Pennisetum* sp.	C3	Stomatal closure	[27]
	0.6 Nm/L; 7 days	*Brassica juncea*	C3	Stomatal closure	[30]
	15 mg/L; 10 days	*Monochoria hastata*	C3	Stomatal closure	[37]
	24 mg/kg; 40 days	*Brassica campestris* ssp. *Brassica juncea* Czernajew	C3	Decreased stomatal conductance	[50]
	0.2 Μm/L; 12 days	*Arachis hypogaea* cv	C3	Pores become smaller	[51]
	50 mg/kg; 10 days	*Glycine max*	C3	Decreased of number stomata	[33]
	5, 10, 30, 50 mg/kg; 7 days	*Zea mays* L.	C4	Increased stomatal conductance (high) Decreased stomatal conductance (low)	[52,53]
	100 mg/kg; 28 days	*Calendula officinalis*	C4	Decreased stomatal conductance	[8]
	10 μM/L; 8 h	*Beta vulgar*	C3	Reduced stomatal aperture and size	[54]
	25, 50 μM/L; 28 days	*Bacopa monniera*		Decreased stomatal conductance	[49]
	42 mg/kg; 10 days	*Hordeum vulgare* L.	C3	Decreased stomatal conductance	[55]
	100 mg/kg; 30, 60, 90 days	*Triticum aestivum* L.	C3	Decreased stomatal conductance	[54]
	10, 50, 100 μM/L; 21 days	*Vigna radiata*	C3	Decreased stomatal conductance	[56]
	25, 100 mg/kg; 60, 90 days	*Cicer arietinum* L.	C3	Decreased stomatal conductance	[38]
	10, 50, 100 μM/L; 20 days	*Cucumis sativus* L.	C3	Decreased stomatal conductance	[57]
	1, 10, 100 μM/L; 15 days	*Hordeum vulgare*	C3	Reducing the density and number of stomata	[58]
	250 μM/L; 90 days	*Schinus molle*		Smaller stomata size	[59]
	7 μM/L; 40 days	*Eichhornia crassipes*	C3	Increased stomatal density	[15]
	30 mg/kg; 42 days	*Melissa officinalis* L.		Reduced stomatal size and reduced stomatal index	[58]
	32 μM/L; 15 days	*Calophyllum brasiliense*		Stomatal closure	[40]
	100 μM/L; 15 days	*Vigna unguiculata* (L.)		Decreased stomatal conductance	[41]
Pb	30.2 mg/kg	*Brassica rapa* spp. pekinensis	C3	Stomatal closure	[60]
	700 μM/L; 21 days	*Leucaena leucocephala* (*Lam*.)	C3	Decreased stomatal conductance	[11]
	1000; mg/kg	*Triticum aestivum* L.	C3	Decreased stomatal conductance	[30]
	0.5, 1, 2, 4 mg/L; 20 days	*Eichhornia crassipes*	C3	Decreased stomatal conductance	[61]
	500 μM/L; 7 days	*Nicotiana tabacum* L.	C3	Stomatal closure	[34]
	500, 1000 mg/kg; 30 days	*Plantago asiatica* L	C3	Decreased stomatal conductance	[62]
	0.5, 1, 2, 4, 8 μM/L; 30 days	*Panicum aquanticum* Poir	C3	Increased stomatal density and Decreased stomatal size	[63]
	40 mg/dm; 14 days	*Glycine max* (Linn.) *Merr*	C3	Stomatal closure	[36]
	1400 mg/kg; 7 days	*Ligustrum lucidum Ait.*		Decreased stomatal conductance	[47]
	300 mg; 21 days	*Lactuca sativa* L.		Decreased stomatal conductance	[64]
Cu	different concentrations in the air	*Gochnatia arequipensis Sandwith*	C3	High stomatal density, small pores.	[65]
	25 mg/L; 10 days	*Vigna mungo* (L.)	*C3*	Stomatal restriction	[33]
	10, 100 mg/kg; 20 days	*Solanum lycopersicum* L.	*C3*	Guard cells are destroyed, stomatal closure	[46]
	2, 20, 200 μM/L; 80 days	*Billbergia zebrina Lindl.*	*C3*	Change in stomata density	[15]
	50 μM/L; 14 days	*Coriandrum sativum*	*C3*	Decreased stomatal conductance	[66]
Zn	1 nM; 25 days	*Populus* × *euramericana*	C3	Changing the number, density of stomata	[64]
	400 μg; 6 months	*Cajanus cajan* (Linn.) Huth	C3	Decreased stomatal conductance	[12]
	5 μM/L; 20 days	*Datura species*	C3	Stomatal closure	[67]
Al	10 mM; 98 days	*Quercus glauca* Thumb	C3	Decreased stomatal conductance	[48]
Cr	150 μM; 20 days	*Zea mays* L.	C4	Decreased stomatal conductance	[68]
Ba	5000 μM; 20 days	*Glycine max*	C3	Stomatal closure	[17]
Ni	100 ppm; 2 years	*Arundo donax* L.	C3	Stomatal conductance declined; increased stomatal resistance	[69]
Hg	1–1000 nM; 49 days	*Picea asperata Mast*	C3	Stomatal closure	[41]
Sb	2000 mg/kg; 60 days	*Acorus calamus* L.	C3	Decreased stomatal conductance	[47]
Ti	20 mg/L	*Quercus ilex subsp* ballota	C3	Decreased stomatal conductance	[70]
As	25 μM; 8 days	*Glycine max*	C3	Decreased stomatal conductance	[33,52]
Mn	150 mg/kg; 15 days	*Brassica juncea* (L.) Czern.	C3	Stomatal closure	[3]
Fe	100 μM/L; 12 days	*Arachis hypogaea* cv	C3	Caused small and abundant stomata on the leaf surface	[52]
Ag	17.7 μM/L; 21 days	*Salix miyabeana*	C3	Decreased stomatal conductance	[71]

### 4.2. The Effect of Heavy Metals on Stomatal Conductance

Stomatal conductance is the degree of stomatal opening, and has a direct effect on plant transpiration. More and more studies have shown that exposure to heavy metals mainly induces the reduction of plant stomatal conductance, but there are also opposing findings. Studies have shown that Cd can cause a significant decrease in the stomatal conductance of *Bacopa monniera*, pakchoi (*Brassica chinensis* L.), mustard (B. nigra), marigolds, Holm oak, mastic shrub, populus, riparian Salix variegata, *Arundo donax* L., cowpea, *Ocimum basilicum* L., *Origanum vulgare* L., and cucumber [4,10,30,50,57,69,70,72,73,74]. Pb can inhibit the stomatal conductance of wheat and plantain [32,62]. Cr can induce the decrease of stomata conductance of corn and sunflower [68,75]; Zn can induce a decrease in the stomatal conductance of cowpea and Datura plants [12,66]. As can induce a significant decrease in soybean stomatal conductance [52], and Cu can induce a decrease in the stomata conductance of coriander [76]. However, other studies have found that Cd can induce increased stomatal conductance in mustard, maize, water hyacinth, and *Lactuca sativa* L. [30,64,77,78], which is probably due to two reasons. First, when heavy metals cause a low concentration of carbon dioxide in plants, the plants can increase their stomatal conductance to obtain more carbon dioxide to meet their respiratory needs and resist the stress of the external environment [64]. Second, the accumulation of heavy metals may lead to a leakage of potassium ions from the plant, thus weakening the plant’s ability to regulate stomatal closure and thus increasing stomatal conductance [64].

### 4.3. The Effect of Heavy Metals on the Amount and Density of Stomata

Changes in the number of stomata are reliable for assessing the level of accumulation and the translocation of heavy metals in plants. An increase in the number of stomata indicates that the enrichment and translocation of heavy metals are occurring within a plant, and the increase may be a way to alleviate heavy metal stress (Figure 3). To maintain the physiological and metabolic functions, plants enhance their heavy metal tolerance by increasing the number of stomata, thereby increasing the surface area of stomata and improving CO_2_ uptake and water availability. Many studies have shown that different heavy metal exposures can increase the number of plant stomata. Specifically, Cd exposure has been shown to induce an increase in the number of stomata in tobacco, shore quinoa, cowpea, and mung bean [56,79]; Pb, Zn, and Cu have been shown to induce an increase in the number of stomata in sunflower [80]; As increases the number of stomata in soybean [33], and Pb causes an increase in the number of stomata in plantain [62]. However, a small number of studies have found that exposure to heavy metals can also lead to reduced stomatal numbers. High levels of exposure to Cu, Cd, and Cr cause a reduction in stomatal numbers in wheat and tomato [81,82,83]. This is likely related to heavy metal concentrations [84]. High heavy metal concentrations can disrupt mitosis in plant cells, leading to damage during cell division, and thereby reducing the number of stomata [85,86].

Stomatal density is also one of the most important indicators for assessing heavy metal stress. Numerous studies have shown that heavy metals cause inconsistent changes in plant stomatal density. For example, Cd stress has been shown to cause a decrease in stomatal density in plants (e.g., Picris) [87]. Low concentrations of lead have been shown to lead to an increase in stomatal density in water hyacinth leaves, while high concentrations of lead have been shown to lead to a decrease in their stomatal density [63]. Cu reduced the stomatal density of Qilian grass [65], and increased the stomatal density of *Billbergia zebrina* (Bromeliaceae) [18]. The main reason for the variation in stomatal density may be related to the compensatory mechanism of plant adaptation to heavy metal stress. An increase in stomatal density and a decrease in stomatal size can reduce the transpiration area and thus avoid excessive water loss. Conversely, a decrease in stomatal density and an increase in stomatal size can maintain CO_2_ flux. In summary, differences in the amount, density, and size of stomata may be an adaptive mechanism of plants to heavy metal stress [67].

### 4.4. The Effect of Heavy Metals on Stomatal Guard Cells

Guard cells are the main component cells of the stomatal complex, and are extremely important for maintaining stomatal function [88,89]. The toxicity of heavy metals to guard cells has therefore attracted much attention. Cd induces a reduction in the length, an increase in the width, and a decrease in the circumference of guard cells in pea plants [90]. Similarly, Pb induces a decrease in the diameter of stomatal guard cells in soybean plants, causing the production of large amounts of starch grains and plastid globules in the guard cell plastids [36]. In addition, Cu can disrupt tomato guard cell membranes, and causes an irregular arrangement of guard cells [45,52]. As can induce a thickening of the cell wall of soybean stomatal guard cells. Pb and Cd can induce ultrastructural changes in rice guard cells, causing a significant distortion and malformation in the shape of guard cells [74]. These results suggest that exposure to different heavy metals can disrupt guard cell morphology and structure, which in turn affects guard cell activity [68].

Whether heavy metal interactions with guard cells are direct or indirect remains controversial. Heavy metals can alter the morphology and structure of guard cells by accumulating in guard cells and interacting directly with intracellular material [41], and Al exposure can lead to the significant accumulation of aluminum in guard cells, which disrupts the cell structure [91]. However, other studies found that the Cd, Cu, and As can cause morphological and structural changes in guard cells, but no accumulation of metals in guard cells has been observed [45,52,92]. In addition, heavy metals can also affect guard cell development by disrupting microtubule tissue, which plays an important role in the development and differentiation of guard cells [93], and can regulate turgor pressure by interfering with potassium channels on guard cell membranes, thereby causing guard cell damage [66].

### 4.5. The Mechanisms of Heavy-Metal-Induced Stomatal Damage

**Figure 4:**
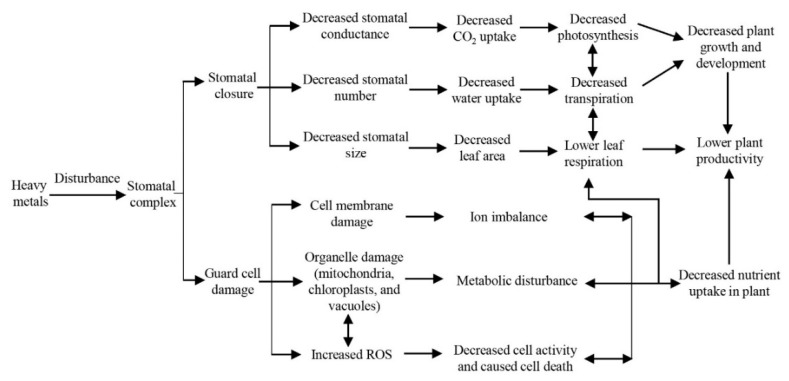
Schematic representation of the effects of heavy metals on plant productivity. This figure summarizes the effect of heavy metals on stomata and ultimately on plant stomata.

Although studies of heavy metal damage to plant stomata have been widely reported, the mechanisms of heavy metal toxicity to stomata are still not fully understood (Figure 4), such as the genetic level, the protein level, and the metabolic level of the stomata. Numerous studies have found that one of the mechanisms of toxicity of heavy metal stress to plants is the generation of oxidative stress (Figure 3) [3,4,5,41]. Excess reactive oxygen species (ROS) accumulate in plant cells under heavy metal stress, thereby causing oxidative stress [46,65,94,95]. ROS are usually produced in plant cell chloroplasts, mitochondria, and subcellular structures, such as peroxisomes, and primarily consist of superoxide anion radicals (O**^−^**^2^), hydrogen peroxide (H_2_O_2_), and hydroxyl radicals (HO) [96]. To counteract oxidative stress, plants adapt to or scavenge ROS by altering the activity of a range of antioxidant enzymes in their bodies [97,98]. Plants catalyze the production of the disproportionation product H_2_O_2_ by O**^−^**^2^·through superoxide dismutase (SOD), followed by the further scavenging of H_2_O_2_ by catalase (CAT) and peroxidase (POD) [96,99]. Low levels of ROSs can act as signalling molecules in plant defense reactions, sending signals to the antioxidant defense system, leading to increased antioxidant enzyme activity [100,101]. However, when ROS levels exceed the scavenging capacity of the antioxidant defense system, oxidative damage to the antioxidant enzymes occurs, leading to a gradual decline in antioxidant enzyme levels and the eventual destruction of the antioxidant enzyme system itself [5,102,103]. Excess ROS in plants can further induce morphological and structural damage to the organism in direct reaction with biomolecules such as lipids, proteins, and nucleic acids [104]. Many studies have shown that Pb and Cd exposure can cause oxidative stress in rice, accompanied by the severe distortion and malformation of the stomatal guard cell shape [105]. Similarly, Cu exposure has been shown to lead to oxidative stress in tomato, accompanied by the destruction of stomatal guard cells [45]. These results further suggest that oxidative stress is likely to be one of the toxic mechanisms underlying stomatal damage induced by heavy metals (Figure 3).

Ion regulation in guard cells may also be a key factor in heavy metal-induced stomatal damage (Figure 3). The accumulation of Pb in soybean guard cells has been found to alter the permeability of the cytoplasmic membrane, which leads to K ion efflux and ultimately to reduced stomatal cell expansion and stomatal closure [36]. The accumulation of Ba metal in soybean guard cells inhibits the translocation of K ions from epidermal cells to guard cells, leading to stomatal closure and ultimately to the inhibition of stomatal photosynthesis and plant productivity [17]. In addition, other studies have shown that Cd can disrupt the Ca channels in Arabidopsis guard cells, thereby affecting stomatal behavior [50]. These results further suggest that ion regulation in guard cells plays an important role in heavy-metal-induced stomatal damage.

## 5. Ecological Damage of Whole Plants in Relation to Stomata Response

Heavy metals are most available in soil and aquatic ecosystems, with only relatively small amounts present in the atmosphere in the form of particles or vapors. There are different sources of heavy metals in the environment, including natural sources, agricultural sources, industrial sources, domestic sewage, and atmospheric sources, among others [2]. Low doses of heavy metals can be attributed to a wide range of activities (mineral development, industrialization, electronic products, transportation, etc.), and the metals are non-degradable and persistent, thereby affecting the survival of plants and the level and pattern of biodiversity.

Stomata (number, behavior, size, and so forth) are the gateway for plants to absorb CO_2_ and affect the plant’s metabolic capacity (e.g., affecting respiration and element uptake) and transpiration. Heavy metal-induced stomatal closure is likely to lead to a reduction in plant survival and reproductive capacity (Figure 4). Survival and reproduction are the main ways in which plants maintain and perpetuate their populations, and changes in survival and reproductive capacity are an important expression of how plants adapt to changes in their environment. Plant survival and reproduction are influenced by many factors, of which water, light, and CO_2_ are the main limiting factors affecting plant growth and reproduction [106]. Numerous studies have shown that plants can modify their ability to survive and reproduce by regulating photosynthesis, transpiration, and respiration. However, changes in the trait function of stomata, the main physiological structures of plants that regulate photosynthesis, in addition to transpiration and respiration, may play a key role in influencing plant survival and reproduction [107]. The closure of plant stomata under drought or environmental stresses leads to reduced CO_2_ fixation and reduced water use by plants, resulting in reduced access to nutrient salts, which in turn leads to a reduction in the survival of plant offspring and ultimately affects plant viability. In contrast, the closure of plant stomata under drought stress or other environmental conditions leads to the reduced transpiration or respiration of plants, resulting in reduced energy storage for reproduction, which reduces plant reproductive capacity [108,109,110,111]. Similarly, under heavy metal stress, plant stomata can close, leading to reduced photosynthesis in plants. For example, it has been found that Cd stress inhibits photosynthesis in plants [51], and Pb stress alters the transpiration rate in tobacco [34]. These findings further suggest that heavy metals can directly or indirectly affect the ability of plants to survive and reproduce by interfering with stomatal activity.

Heavy metals interfere with the plant metabolism, resulting in the inhibition of plant growth and yield. The productivity of plants depends on their growth and development. There are various functional roles undertaken by ecosystems, which are mainly expressed in terms of productivity, energy flow, material cycling, and information transfer [110,112]. Pollutant-induced phytotoxicity impairs the function and efficiency of the photosynthetic system, stomatal function, and cambium activity. Finally, pollution stress affects the biochemical parameters of plants and inhibits the ability of plants to perform physiological functions such as photosynthesis, transpiration, and respiration. The toxic effects caused by pollutants can lead to significant plant damage and reduced growth and yield, ultimately affecting plant productivity [113]. Plants are the primary producers of ecosystems, and their functional traits are closely related to changes in ecosystem function. Plant functional traits are those that respond to changes in the living environment or have an impact on ecosystem function, and mainly include structural traits (e.g., leaf area, stomatal density, and stomatal conductance) and physiological traits (e.g., the leaf photosynthetic rate and the water use rate) [114,115,116,117]. Studies have confirmed that environmental changes can cause changes in plant functional traits, and thus in ecosystem function [118,119]. The efficiency of light energy utilization is a key indicator for plants to convert energy intercepted from the environment into organic matter through photosynthesis. It is also a key factor influencing the productivity, capacity, and quality of ecosystems [120,121]. Studies have shown that under drought conditions, a decrease or increase in the number of stomata leads to an increase and decrease in the leaf area, resulting in a decrease in plant primary productivity [109]. Under drought stress, the closure of stomata causes a blockage of plant respiration, water and nutrient uptake, thus affecting plant material and energy cycling processes [122]; Under drought stress, stomatal closure leads to the reduced exchange of information material inside and outside the plant, resulting in blocked information transfer [123,124]. However, an increasing number of studies have shown that under heavy metal stress, Cu exposure leads to a reduction in plant stomatal numbers or stomatal conductance or density, resulting in a reduction in the leaf area, which may cause a reduction in plant productivity [65]; Cd exposure leads to the closure of plant stomata, resulting in reduced photosynthetic or respiratory rates or water use rates, which may cause impaired material and energy cycling [42]; Studies have shown that the poisoning of phytoplankton by high concentrations of heavy metals (Cu, Cr, Pb, etc.) reduces stomatal opening and stomatal conductivity, especially the poisoning and inactivation of enzyme systems, seriously affecting physiological and biochemical processes such as photosynthesis, respiration, protein synthesis and cellular organic matter synthesis, which may cause impaired information transfer [9,53,125]. These results further suggest that heavy metals affect ecosystem function by altering the physiological ecology of stomata, thereby causing changes in structural (e.g., leaf area, stomatal density and stomatal conductance) and physiological (e.g., leaf photosynthetic rate and water use rate) traits in plants (Figure 4).

## 6. Conclusions and Future Perspectives

This paper reviewed a range of biotoxic effects and mechanisms of heavy metal stress on plant stomata. It was found that the effects occurred primarily by altering stomatal behavior and disrupting the morphology, structure, and function of stomatal-associated cells. Stomata can enhance heavy metal tolerance by altering uptake behavior and regulating cell morphology and structure. Oxidative stress is a main mechanism by which heavy metals induce stomatal damage. Although the effects of heavy metals on stomata have been reviewed from several perspectives in this paper, there are some limitations. Previous studies have shown that stomatal function was regulated by various genes (e.g., stomatal closure-related actin binding protein 1 (*SCAB1*), actin 2 (*ACT2*) and myosin (*MYOATP*)), and that the differential expression of these genes can cause changes in stomatal function [126,127]. Unfortunately, studies on the effects of heavy metals on specific stomatal genes are very few in number [128]. Therefore, the mechanisms of the effects of heavy metals on stomatal functions (e.g., water utilization) have not been explored in depth in this paper.

Previous studies have primarily focused on the effects of heavy metals on stomatal behavior, morphology, and structure, while the mechanisms of the toxicity of heavy metals on stomata are still unclear. Studies have focused on characterizing the toxic endpoints of stomata under heavy metal stress, while there have been a few studies of the mechanisms of heavy-metal-induced stomatal development. With the continuous development of omics technology, the molecular level of stomata under heavy metal stress (genes, proteins, and metabolites) will be explored in greater depth. Unfortunately, there have been very few studies of the impact of heavy metals on stomata at the molecular level. To better understand the stomatal response to heavy metals, a combination of multiple omics technologies is needed to comprehensively profile the changes in stomata at the molecular level.

Most studies have used acute short-term exposure experiments with high concentrations of heavy metals in laboratories or greenhouses rather than investigations under natural conditions. Low doses of heavy metals can be attributed to a wide range of activities, and the metals are invisible and persistent, with the potential to become pollutants. Most heavy metals do not exist alone in soils but are present with other metals, which can affect the level of plant survival and biodiversity. Given the realistic concentrations of heavy metals in actual soils, there is an urgent need to conduct long-term soil exposure experiments with environmentally relevant concentrations of heavy metals to obtain more scientifically valid data. Given the multiple functions of stomata in plant physiology, damaged stomata can alter plant photosynthesis, transpiration, water use, and even plant metabolism, ultimately affecting plant growth and development. A key goal of future research in this field is to determine the toxicity mechanism of the interaction between heavy metals and stomata and revealing the mechanism of heavy metal effects on stomata can provide a scientific basis for assessing the contamination risk of soil heavy metals. In future studies, exogenous hormones, PGPR, and melatonin can be used to overcome heavy metal stress and its influence on physiological behavior.

## Data Availability

Not applicable.
